# Extracellular histones play a pathogenic role in primary graft dysfunction after human lung transplantation

**DOI:** 10.1039/d0ra00127a

**Published:** 2020-03-27

**Authors:** Yang Jin, Meng Sun, Xin Lv, Xingan Wang, Gening Jiang, Chang Chen, Zongmei Wen

**Affiliations:** Department of Anesthesiology, Shanghai Pulmonary Hospital, Tongji University School of Medicine Zhengmin Road 507 Shanghai 200433 China wzm1103@126.com; Division of Pulmonary, Allergy, and Critical Care Medicine, University of Pittsburgh School of Medicine Pittsburgh PA 15213 USA; Department of Thoracic Surgery, Shanghai Pulmonary Hospital, Tongji University School of Medicine Shanghai 200433 China chenthoracic@163.com

## Abstract

Primary graft dysfunction (PGD) causes early mortality and late graft failure after lung transplantation. The mechanisms of PGD are not fully understood but ischemia/reperfusion (I/R) injury may be involved. Extracellular histones have recently been identified as major contributors to I/R injury. Hence, we investigated whether extracellular histones are associated with PGD after lung transplantation. In total, 65 lung transplant patients were enrolled into this study. Blood samples were collected from patients before and serially after transplantation (24 h, 48 h, and 72 h) and measured for extracellular histones, myeloperoxidase (MPO), lactate dehydrogenase (LDH), and multiple cytokines. Besides, the patients' sera were cultured with human pulmonary artery endothelial cells (HPAEC) and human monocyte cell line (THP1) cells, respectively, and cellular viability and cytokine production were determined. Heparin or anti-histone antibody were used to study the effects of histone-neutralized interventions. The results showed that extracellular histones increased markedly after lung transplantation, peaked by 24 h and tended to decrease thereafter, but still retained high levels up to 72 h. Extracellular histones were more abundant in patients with PGD (*n* = 8) than patients without PGD (*n* = 57) and linearly correlated with MPO, LDH, and most detected cytokines. *Ex vivo* studies showed that the patients' sera collected within 24 h after transplantation were very damaging to HPAEC cells and promoted cytokine production in cultured THP1 cells, which could be largely prevented by heparin or anti-histone antibodies. These data suggested a pathogenic role for extracellular histones in PGD after lung transplantation. Targeting extracellular histones may serve as a preventive and therapeutic strategy for PGD following lung transplantation.

## Abstract 2

Primary graft dysfunction (PGD) causes early mortality and late graft failure after lung transplantation.

## Graphical abstract

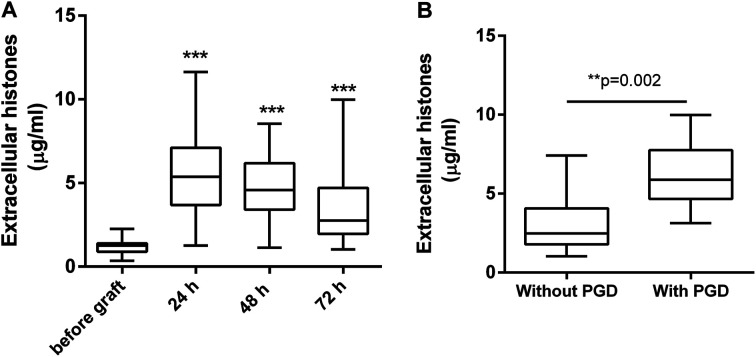

## 1. Introduction

Lung transplantation is now considered the most effective treatment for end-stage lung diseases.^1^ With the improvement of preservation methods and surgical techniques, as well as the use of immunosuppressants, lung transplantation outcomes have significantly improved in the past decades.^2^ However, because of the special anatomical structure and physiological functions of the lung, several complications such as primary graft dysfunction (PGD) are commonly seen after lung transplantation, which may be the reason that survival after lung transplantation is relatively low compared with other organ transplants.^3,4^ PGD is a form of acute lung injury (ALI) that develops within the first 72 hours after lung transplantation.^5^ It is defined by the presence of hypoxemia and radiographic infiltrates, and is a major cause of early mortality and morbidity after transplant.^6^ The incidence of PGD after lung transplantation is estimated to be 10 to 30%.^3^ PGD is also associated with the development of bronchiolitis obliterans syndrome (BOS), a form of chronic lung allograft dysfunction.^7^ Patients with PGD usually had prolonged hospitalization, and increased short- and long-term mortality when compared to non-PGD patients.^8^ So far, no specific therapies are available for PGD.

The pathogenesis of PGD is multifactorial but ischemia-reperfusion (I/R) injury is commonly implicated, which may play a central role resulting in transplant lung dysfunction.^9^ Lung I/R injury is a complex pathophysiologic process involving many types of cells and mediators. Of these, extracellular histones have been considered as key mediators implicated in I/R injuries of many organs including the lung, liver, kidney, heart, and brain.^10–13^ Extracellular histones increase abundantly after I/R injury, which are not only correlated with disease severity and poor outcomes, but also may act as a potential therapeutic target.^14–16^ It has been confirmed that targeting extracellular histones ameliorates the I/R injuries of many organs.^17^ In view of the critical role of extracellular histones in mediating I/R injury, we aimed to investigate whether extracellular histones may play a pathological and targetable role in PGD after human lung transplantation.

## 2. Patients and methods

### 2.1. Patients

Totally 65 patients undergoing lung transplantation between August 2015 and October 2019 were included in this study. The study was approved by the Institutional Review Board of Shanghai Pulmonary Hospital, Shanghai, China and performed with the Declaration of Helsinki. All patients or their nominated next of kin provided written informed consent. Baseline characteristics of these patients were recorded. PGD is defined by radiographic presence of diffuse pulmonary infiltrates within 72 hours after lung transplantation.^5^ Clinical manifestations of PGD are similar to non-cardiogenic pulmonary edema, featured as hypoxemia, hypotension and low cardiac output caused by decreased lung compliance and increased pulmonary capillary resistance.^18^

### 2.2. Blood sample collection

The peripheral blood samples from patients were collected before and serially after lung transplantation. A total of 4 time points was defined: before graft, within 24 hours, 48 hours, and 72 hours after graft. Blood samples were collected into citrated vacutainers and centrifuged at 2000 × *g* for 20 min and the generated plasma was separated, aliquoted and stored at −80 °C. The matched plasma was also produced for another set of *ex vivo* experiments.

### 2.3. Measurement of extracellular histones and multiple cytokines

The levels of extracellular histones (nucleosomes) in the plasma samples were determined using a Cell Death Detection ELISA kit (Roche, Germany).^19^ The purified calf thymus histones were used to generate standard curves. For detection of various cytokines (IL-1β, IL-2, IL-4, IL-5, IL-6, IL-8, IL-10, IL-12p70, IL-17A, IL-18, IL-22, IL-23, IL-27, GM-CSF, TNF-α), a panel of multiplex immunoassay from Affymetrix eBioscience (USA) was used on a Luminex/Bioplex-200 system.

### 2.4. Measurement of MPO and LDH

Plasma MPO, a classical marker for neutrophil and monocyte/macrophage activation, and LDH, a cytoplasmic enzyme indicating cellular damage, were measured using commercial kits (Roche, Germany), according to the manufacturer's protocols.^20^

### 2.5. *Ex vivo* experiments

Human pulmonary artery endothelial cells (HPAEC) and human monocyte cell line (THP1) were obtained from American Type Culture Collection (ATCC). 2 × 10^5^ cells were seeded in 24-well plates and cultured in RPMI-1640 (10% fetal bovine serum, 100 U ml^−1^ penicillin/streptomycin, 2 mM glutamine) in a 5% CO_2_ humidified atmosphere at 37 °C. After the cells reached 80–90% confluence, they were incubated with 50% of the sera samples (approximately 400 μl) for 24 h, which were pooled from the patients before or within 24 h after transplantation, respectively. To verify the impact of extracellular histones in the sera, 20 μg ml^−1^ of anti-histone H4 antibody or 200 U ml^−1^ of heparin (Sigma-Aldrich, USA) was added to the cultured cells, respectively.

For the cytotoxicity assay, the cultured HPAEC cells were detached and washed with PBS and incubated with PI dye solution (10 μg ml^−1^, Sigma-Aldrich, USA) in the dark for 10 min at room temperature. The cells were then subjected to flow cytometry analysis. In addition, the supernatants were collected and analyzed for LDH levels. To test the influence of extracellular histones in sera samples on THP1 cells, the supernatants (approximately 100 μl) were collected and analyzed for the production of multiple cytokines (IL-1β, IL-6, IL-10, IL-17A, IL-18, TNF-α) using a Multiplex Immunoassay kit from Affymetrix eBioscience.

### 2.6. Statistical analysis

For human data, values were presented as medians and interquartile ranges. For *ex vivo* data, values were presented as mean ± standard deviation (SD). The results were analyzed using unpaired Student's *t* test or Mann–Whitney test (for two groups), one-way ANOVA with Turkey post-tests (for more than two groups). The correlations were analyzed using Spearman's correlation or Pearson correlation analysis. Differences were considered statistically significant when *p* < 0.05. All data were analyzed using GraphPad Prism (GraphPad Software, Inc., San Diego, CA, USA).

## 3. Results

### 3.1. Patient characteristics

A total of 65 patients received lung transplantation. The baseline characteristics of the patients were indicated in Table 1. Main reasons for transplantation were COPD (n = 23, 35.38%), interstitial lung disease (n = 20, 30.67%), idiopathic pulmonary fibrosis (n = 12, 18.46%), pulmonary hypertension (n = 2, 3.07%) and others (n = 8, 12.3%). Donor lungs were obtained from deceased cardiac failure patients.

**Table 1:** Baseline characteristics in patients undergoing lung transplantation^a^

Characteristics	Values
Age (years)	60.60 ± 11.17
Gender (male/female)	58/7
BMI	21.37 ± 3.76
Operation time (min)	239.1 ± 71.79

**Etiology of lung diseases**	
Chronic obstructive pulmonary disease	23 (35.38%)
Idiopathic pulmonary fibrosis	20 (30. 67%)
Interstitial lung disease	12 (18.46%)
Pulmonary hypertension	2 (3.07%)
Others	8 (12.3%)
Hospital stay (day)	36 (28–60)

**Type of transplant**	
Double lung transplant	5 (7.69%)
Single lung transplant	60 (92.31%)

a Data were expressed as mean (SD) or number of patients (%).

### 3.2. Extracellular histones increased significantly after lung transplantation

**Fig. 1:**
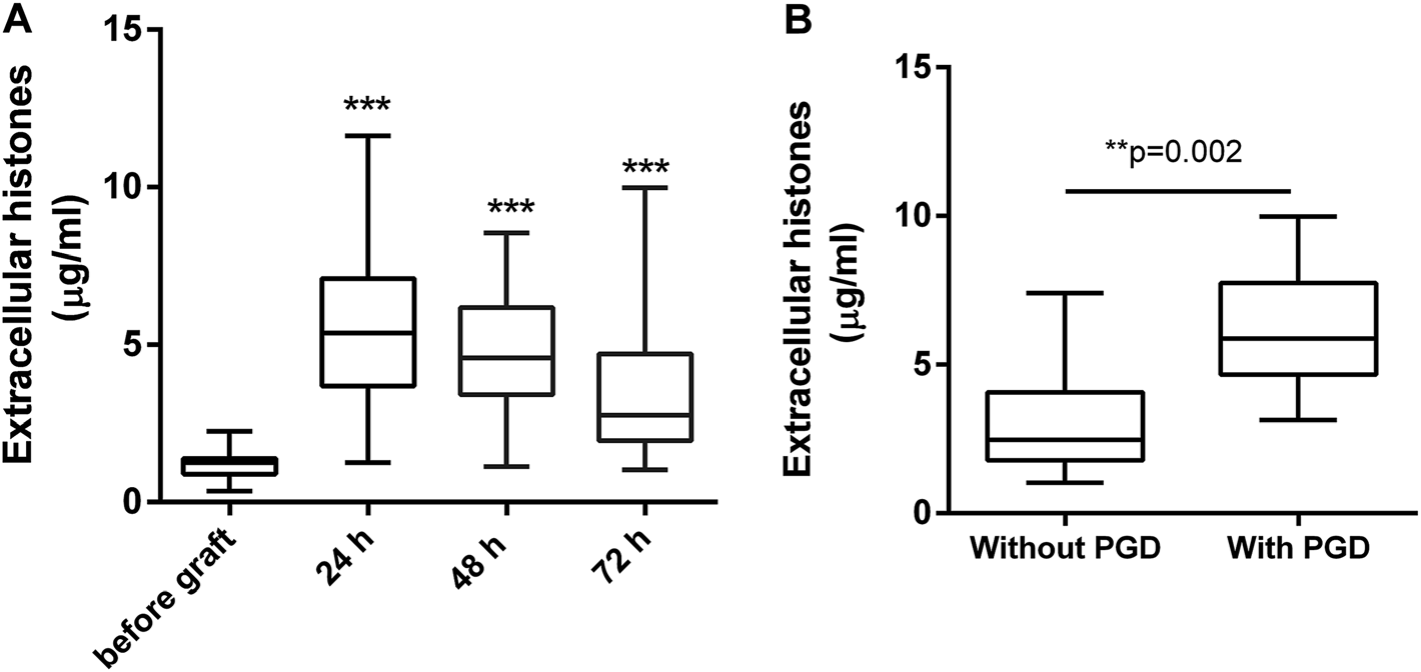
The levels of extracellular histones in the blood of patients undergoing lung transplantation. (A) Extracellular histone levels were increased significantly in patients undergoing lung transplantation at different time points. (B) Median extracellular histones at 72 h after graft were significantly higher in patients with PGD than those in patient without PGD. Values were presented as median (interquartile range).

Extracellular histone levels in the patients following lung transplantation were 4–6 times higher than the levels measured before transplantation. Extracellular histones reached a peak by 24 h after graft [5.368 (3.686, 7.103) μg ml^−1^] and then gradually decreased within 72 h [2.756 (1.961, 4.713) μg ml^−1^] but were still higher than the levels before transplantation [1.251 (0.896, 1.398) μg ml^−1^] (Fig. 1A). Based on an early evaluation (within 72 h after graft), the patients were divided into the groups of with PGD (*n* = 8) and without PGD (*n* = 57). Extracellular histones in patients with PGD were significantly higher than that in the patients without PGD [5.882 (4.664, 7.755) *vs.* 2.478 (1.789, 4.073) μg ml^−1^] (Fig. 1B).

### 3.3. Extracellular histones correlated with increased MPO, LDH, and systemic inflammation

**Fig. 2:**
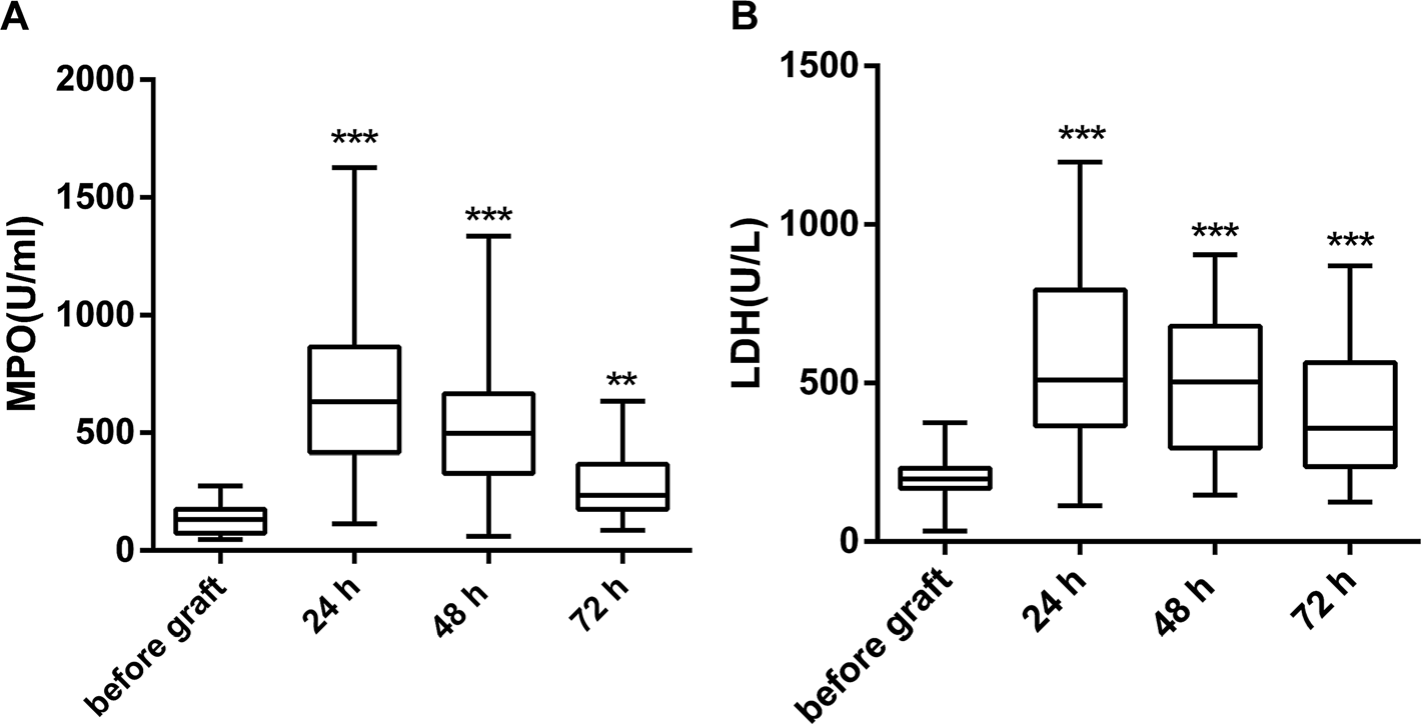
The levels of MPO and LDH in the blood of patients undergoing lung transplantation. (A) MPO (B) LDH levels were increased markedly at 24 hours after graft and showed a decreasing trend at 48 h and 72 h after graft but were still significantly higher as compared with the levels before graft. Values were presented as median (interquartile range).

When compared with the levels measured before graft, both MPO and LDH were significantly elevated by 24 h after graft. They showed a decreasing trend in the following time points (48 h, 72 h) but were still higher than the levels before graft (Fig. 2). The changes in extracellular histones correlated with the changes in MPO and LDH (Table 2).

**Table 2:** Correlation of extracellular histones with various variables in patients undergoing lung transplantation^a^

	*r*	*p*
MPO	0.4369	0.011*
LDH	0.5292	0.007*
IL-1β	0.3023	0.064
IL-6	0.5047	0.007*
IL-10	0.6915	0.001*
IL-17A	0.3899	0.024*
IL-18	0.5176	0.008*
TNF-α	0.3792	0.012*

a *p < 0.05 was considered to be statistically significant.

**Fig. 3:**
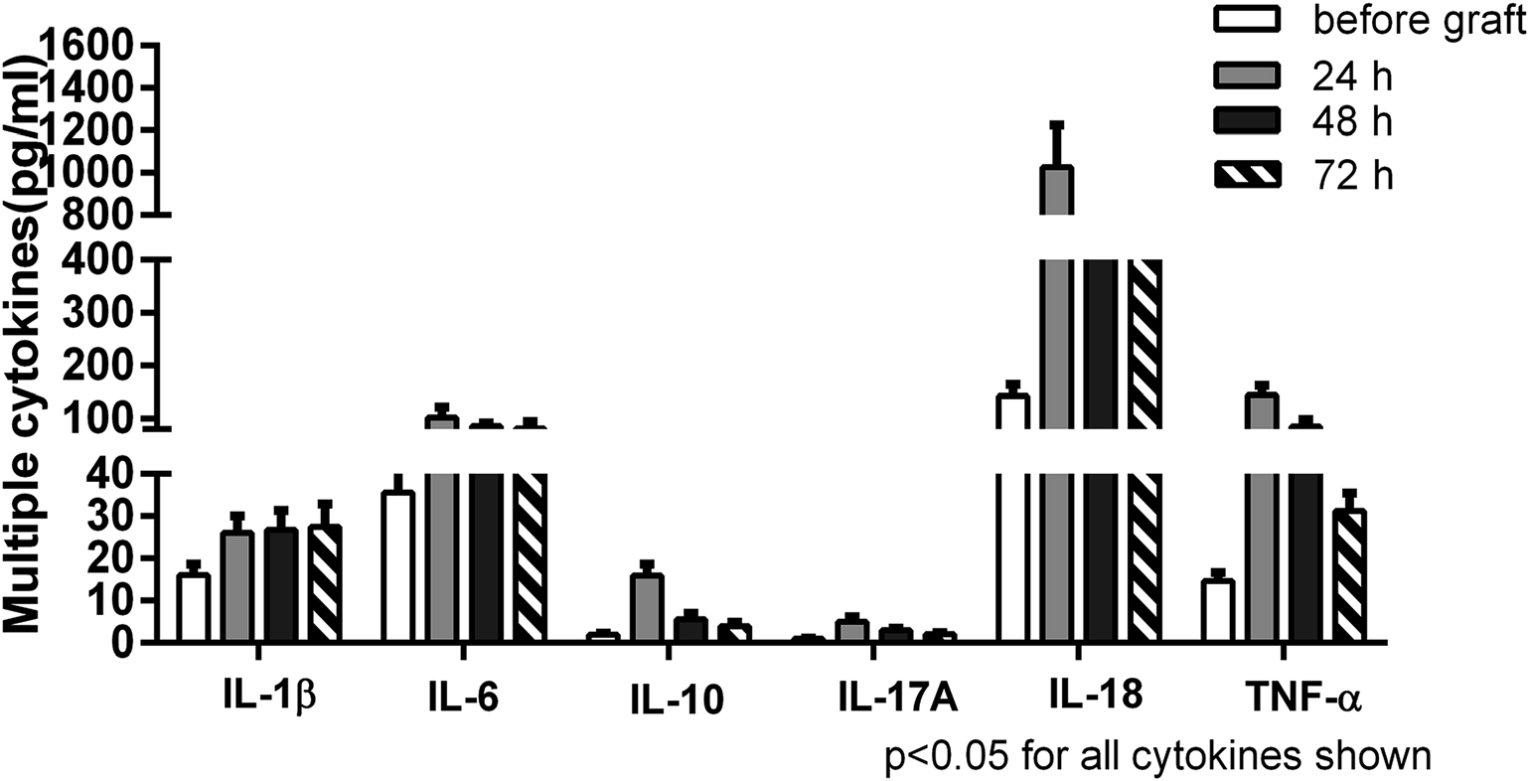
Detection of systemic inflammation in lung transplantation patients. Multiplex immunoassay for a panel of various cytokines (IL-1β, IL-2, IL-4, IL-5, IL-6, IL-8, IL-10, IL-12p70, IL-17A, IL-18, IL-22, IL-23, IL-27, GM-CSF, TNF-α) was performed. Only 6 cytokines with significant differences (*p < 0.05 in contrast to before graft) were shown. Values were presented as median (interquartile range).

A panel of multiple cytokines was measured as well to assess the degree of systemic inflammation. Of these, a total of 6 cytokines (IL-1β, IL-6, IL-10, IL-17A, IL-18, TNF-α) were significantly increased in the patients by 24 h after lung transplantation in contrast to their levels measured before graft. Subsequently, most cytokines exhibited a decreasing trend but remained high levels up to 72 h. Extracellular histone levels were positively correlated with most detected cytokines (Table 2). Taken together, these data suggested that the increased extracellular histones might play a pivotal role in promoting innate immune cell activation, cellular damage, and the enhanced systemic inflammation after lung transplantation (Fig. 3).

### 3.4. Extracellular histones provoked cytotoxicity and cytokine production *in vitro*

**Fig. 4:**
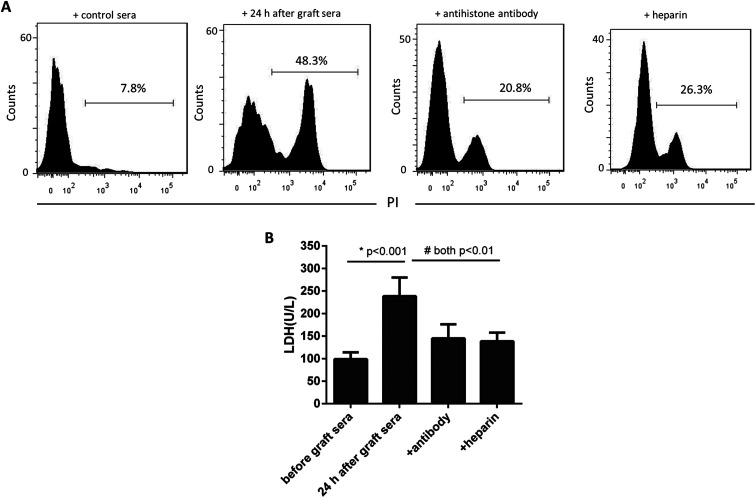
Lung transplantation patients' sera induced direct cytotoxicity towards HPAEC cells. (A) Flow cytometric analysis of PI staining showed that the patients' sera collected at 24 h after graft that contained high levels of extracellular histones were injurious to cultured HPAEC cells. (B) The LDH levels in the supernatants of cultured HPAEC cells treated with the sera pooled at 24 h after graft were significantly increased. Administration of anti-histone H4 antibody or heparin equally improved cell damage. Variables were expressed as mean ± standard deviation (SD).

We next asked whether the increased extracellular histones in patients' blood may have adverse effects contributing to lung injury in PGD or other complications. The patients' sera collected at 24 h after graft were found to contain the highest levels of extracellular histones (median value, 5.368 μg ml^−1^) and thereby pooled and incubated with HPAEC and THP1 cells, respectively. The sera before graft (with histone median value of 1.251 μg ml^−1^) were also included with the cells as the controls. As shown in Fig. 4, the patients' sera at 24 h after graft significantly reduced HPAEC cell viability and promoted production of various inflammatory cytokines in the supernatant of cultured THP1 cells.

**Fig. 5:**
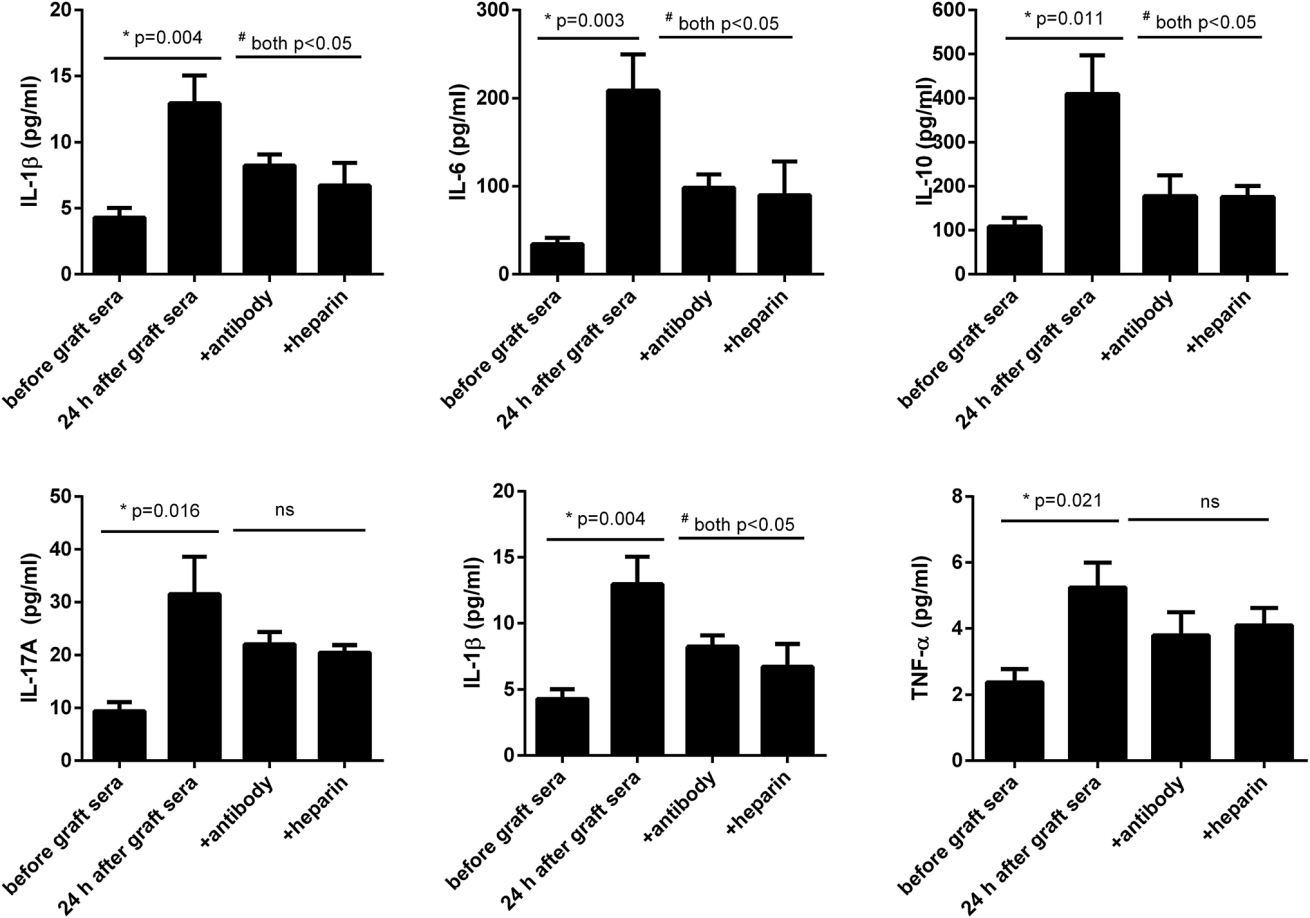
Lung transplantation patients' sera provoked human monocytic cells. It showed that 6 histone-related cytokines were all increased in the supernatants of human monocytic THP1 cells treated by lung transplantation patients' sera collected at 24 h after graft, whereas addition of anti-histone H4 antibody or heparin largely decreased these cytokine levels. Variables were expressed as mean ± standard deviation (SD).

Neutralization of extracellular histones has been reported to be protective in various inflammatory conditions.^21,22^ Here we investigated whether heparin, which is able to bind histones,^23^ would diminish the observed effects of histones on the cells. Anti-histone H4 antibody was applied as the control. The results showed that administration of heparin or anti-histone antibody equally inhibited the patients' sera-induced HPAEC damage and cytokine production from THP1 cells, as evidenced by improved cell viability and decreased inflammatory cytokine levels (Fig. 4 and 5). Collectively these data suggest a direct relationship between extracellular histones and cellular damage and systemic inflammation, and provide a possible therapeutic target in clinical management of PGD after lung transplantation.

## 4. Discussion

Lung transplantation has been proved to be the most effective life-saving therapy for the end-stage lung disease patients such as chronic obstructive pulmonary disease (COPD), interstitial lung disease, idiopathic pulmonary fibrosis, and pulmonary hypertension.^1^ Currently, the prognosis of patients undergoing lung transplantation has significantly improved because of the development of surgical techniques and wide administration of immunosuppressants.^2^ However, PGD is still one of the most common and life-threatening complications affecting lung transplantation outcome. PGD occurs in up to 30% of lung transplant patients within the first 72 hours after graft, characterized by the development of bilateral pulmonary infiltrates and noncardiogenic pulmonary edema.^24^ Discouragingly, there are no certified effective methods for the prevention and treatment of PGD. There were many risk factors for PGD including surgical risk factors, donor-related factors and recipient-related factors.^2^ Among these factors, I/R injury is considered to be the most common and critical.^25^ In the process of I/R injury, various inflammatory mediators and cytokines released abundantly, which further trigger inflammatory response cascade and lead to the damage of vascular endothelium and alveolar epithelial cells.^25–27^ Notably, extracellular histones are discovered to be key mediators of I/R injury and targeting extracellular histones may attenuate I/R-related cell and tissue damage.^28^ Hence, we intended to investigate whether extracellular histones may contribute to the occurrence of PGD in human lung transplantation.

Histones, including H1, H2A, H2B, H3, and H4, have been a traditional research focus in the context of regulating nuclear architecture.^29^ Under conditions of cellular stress or injury such as trauma, ischemic or chemical injury, or infection, histones can be released from both dying cells (*e.g.*, apoptotic or necrotic cells) and activated immune cells (*e.g.*, neutrophils) into the extracellular space.^11^ It has revealed that extracellular histones have the ability to cause endothelial cytotoxicity, platelet activation and aggregation, and most importantly act as danger/damage-associated molecular patterns (DAMPs) to initiate and promote systemic inflammation.^11,30^ The link between extracellular histones and I/R injury has been recognized;^14^ however, it is uncertain whether extracellular histones are associated with PGD in human lung transplantation. Our hypothesis was that extracellular histones are a danger signal contributing to PGD pathogenesis.

In this study, we determined extracellular histone levels in patients undergoing lung transplantation and found that extracellular histones were elevated abundantly after transplantation and peaked by 24 h with high levels still measurable through 72 h. Previous studies showed that I/R injury of various organs consistently caused an elevation of extracellular histones.^31^ Thus, the observed increase in the extracellular histones in this study is likely derived from I/R injury during the process of transplantation. It is noticeable that, compared with patients without PGD, patients with PGD had significantly higher levels of extracellular histones after graft, thereby suggesting that extracellular histones may act as an important cause for PGD. In addition, we detected an evident cellular damage and pronounced immune cell activation, along with a significant inflammatory response after lung transplantation. Extracellular histones correlated with cellular damage, immune cell activation, and systemic inflammation, all of these are in line with previous studies that extracellular histones may induce cytotoxicity and act as a DAMP molecule to enhance systemic inflammation.^32,33^

To further confirm these findings, we incubated the patients' sera collected after graft and contained higher levels of extracellular histones with HPAEC and THP1 cells and observed that the sera induced massive HPAEC damage as well as caused significant production of inflammatory cytokines from cultured THP1 cells. We further confirmed that those effects on cell death and cytokine production were caused from histones, since addition of specific anti-histone H4 antibody and heparin into the cells significantly prevented HPAEC death and production of cytokines from THP1. These data suggest that the elevated extracellular histones is an important determinant for the development of PGD after transplantation and blockade of extracellular histones may serve as a preventive and therapeutic strategy for PGD following lung transplantation.

In summary, our data provide a plausible explanation for the mechanisms related to PGD after lung transplantation. Lung transplantation-related I/R injury causes damage of cells and tissues, and leads to the release of histones from these dying cells into extracellular space such as the circulation. High concentrations of extracellular histones induce direct cytotoxicity, as well as act as DAMP molecules to aggravate systemic inflammation by stimulating immune cells to produce multiple cytokines, which eventually contribute to PGD or other complications. Therefore, determining extracellular histone levels may predict the occurrence of early graft dysfunction in human lung transplantation. More importantly, extracellular histone-targeted therapy appears to be potentially effective for clinical management of PGD, which requires more investigations in the future.

## Conflicts of interest

The authors declare that they have no conflict of interests.

## Supplementary Material
